# Engineering the l-Arabinose Isomerase from *Enterococcus Faecium* for d-Tagatose Synthesis

**DOI:** 10.3390/molecules22122164

**Published:** 2017-12-06

**Authors:** Marylane de Sousa, Ricardo M. Manzo, José L. García, Enrique J. Mammarella, Luciana R. B. Gonçalves, Benevides C. Pessela

**Affiliations:** 1 Department of Chemical Engineering, Federal University of Ceará, Campus do Pici, BL 709, Fortaleza-CE 60455-760, Brazil; marylaneufc@gmail.com; 2 Food and Biotechnology Engineering Group, Institute of Technological Development for the Chemical Industry, National University of the Litoral (UNL), National Council of Scientific and Technical Research (CONICET), RN 168 Km 472 “Paraje El Pozo” S/N, S3000 Santa Fe, Argentina; manzoricardo@yahoo.com.ar (R.M.M.); ejoma@intec.unl.edu.ar (E.J.M.); 3 Center for Biological Research, CIB, Higher Council for Scientific Research, CSIC, C/Ramiro de Maeztu, 9, 28040 Madrid, Spain; jlgarcia@cib.csic.es; 4 Department of Food Biotechnology and Microbiology, Institute of Research in Food Sciences, CIAL, Higher Council for Scientific Research, CSIC, C/Nicolás Cabrera 9, UAM Campus, 28049 Madrid, Spain; 5 Department of Engineering and Technology, Polytechnic Institute of Sciences and Technology, Av. Luanda Sul, Rua Lateral Via S10, P.O. Box 1316, Talatona-Luanda Sul, Angola

**Keywords:** l-arabinose isomerase, recombinant DNA, affinity purification, d-tagatose, d-galactose

## Abstract

l-Arabinose isomerase (EC 5.3.1.4) (l-AI) from *Enterococcus faecium DBFIQ* E36 was overproduced in *Escherichia coli* by designing a codon-optimized synthetic *araA* gene. Using this optimized gene, two N- and C-terminal His-tagged-l-AI proteins were produced. The cloning of the two chimeric genes into regulated expression vectors resulted in the production of high amounts of recombinant *N*-His-l-AI and *C*-His-l-AI in soluble and active forms. Both His-tagged enzymes were purified in a single step through metal-affinity chromatography and showed different kinetic and structural characteristics. Analytical ultracentrifugation revealed that *C*-His-l-AI was preferentially hexameric in solution, whereas *N*-His-l-AI was mainly monomeric. The specific activity of the *N*-His-l-AI at acidic pH was higher than that of *C*-His-l-AI and showed a maximum bioconversion yield of 26% at 50 °C for d-tagatose biosynthesis, with Km and Vmax parameters of 252 mM and 0.092 U mg^−1^, respectively. However, *C*-His-l-AI was more active and stable at alkaline pH than *N*-His-l-AI. *N*-His-l-AI follows a Michaelis-Menten kinetic, whereas *C*-His-l-AI fitted to a sigmoidal saturation curve.

## 1. Introduction

There are diverse varieties of sweeteners that have been developed as reduced-calorie replacements for sucrose in foods [1]. d-Tagatose, a natural ketohexose recognized and classified as a GRAS (Generally Recognized As Safe) substance [2], is a promising sweetener due to its beneficial functional properties [3]. Nevertheless, although d-tagatose has shown great potential for its application in foods and beverages [4,5], its use remains limited owing to its high cost [6].

The production of d-tagatose from d-galactose through enzymatic synthesis is attractive as an industrial process [7,8] when compared with the chemical conversion process, i.e., mild reaction conditions, no by-products, a lack of salt in the produced wastewater, and the possibility of recycling the unreacted d-galactose [9]. l-Arabinose isomerase (EC 5.3.1.4) (l-AI) encoded by *araA*-like genes has been the most studied enzyme for the conversion of d-galactose to d-tagatose due to the industrial viability of this process, since d-galactose can be obtained from the lactose contained in cheese whey as a cheap substrate [10,11]. For this reason, the production of d-tagatose from d-galactose using l-AI has been studied in recent years [6,11,12,13]. However, the enzymatic process still has a number of bottlenecks that need to be overcome, e.g., low protein concentration in the fermentation step using wild-type-producing strains [14,15], low productivity in the isomerization reaction, and reduced thermostability of the enzyme [16,17].

The production of l-AI has been reported by using wild type strains, e.g., *Lactobacillus* [18,19] or *Enterococcus* [11], as well as some recombinant microorganisms, e.g., *Escherichia coli* [10,20] *Bacillus subtilis* [21], or *Lactobacillus plantarum* [22]. In some cases, the recombinant l-AI has been fused to a His-tag to facilitate its purification (e.g., C-terminal His-tag fusion of l-AI from *Thermotoga maritime* [23] or *Thermoanaerobacterium saccharolyticum* NTOU1 [12] and N-terminal His-tag fusion of l-AI from *Bacillus coagulans* [24], *Lactobacillus reuteri* [22], or *Thermus * sp. [25]). However, how the presence of His-tag in the N- or C-terminal ends of the l-AI influences its activity has been investigated only in the l-AI from *Pediococcus pentosaceus* PC-5, and, surprisingly, the His-tag inactivated the enzyme [13]. The production of a recombinant l-AI represents a technological advantage not only because of the high production yield that can be obtained, but also of the possibility of modifying the enzyme by genetic engineering techniques [26]. Nevertheless, none of the *araA* genes expressed in heterologous bacterial hosts have been chemically synthesized and host-codon-optimized to increase the enzyme production so far.

The present work deals with the overproduction of a recombinant l-AI in *E. coli* using the codon-optimized synthetic *araA* gene of *Enterococcus faecium*. Two recombinant l-AIs were designed carrying a 6-His-tag fused at either the C- or N-terminal ends. The chimeric His-tagged-l-AIs were purified by affinity chromatography in a single step and the most relevant biochemical properties (activity, stability, and kinetic parameters) of these novel chimeric enzymes were assessed.

## 2. Results and Discussion

### 2.1. Design of the Recombinant l-AIs for Their Overproduction in E. coli

The *araA* gene from *E. faecium* DBFIQ E36 was selected for producing l-AI since this organism has been identified as an interesting source of the enzyme for the transformation of d-galatose into d-tagatose [15]. Taking into account that the production of l-AI in the native host was low and not suitable for industrial purposes, we decided to overproduce its l-AI in *E. coli*. As described in the Materials and Methods, we created a new synthetic *araA* gene with optimized codons to achieve an optimal expression in the heterologous *E. coli* host. Moreover, we added to the synthetic gene six histidine codons either to the 5′ or to the 3′ end of the gene in order to facilitate the purification of the resulting N- and C-terminal His-tagged l-AI proteins by affinity chromatography. We have tested both His-tagging options to investigate their effects on the enzyme properties in order to select the most efficient chimeric enzyme.

We also decided to test the production of the recombinant enzymes by using different configurations, i.e., using two alternative strong inducible promoters such as ParaC and PT7 in two different hosts, such as *E. coli* DH10B and BL21 (DE3), respectively. Therefore, the l-AI recombinant chimeric protein containing the His-tag at its N-terminal was produced using pBAD-ARA plasmid, a derivative of the pBAD/HisA vector that provides a ParaC promoter inducible by l-arabinose and the resistance to ampicillin. By contrast, the l-AI recombinant protein containing the His-tag at its C-terminal was produced using pET29-ARA plasmid, a derivative of the pET29a vector that provides a PT7 promoter inducible by IPTG (Isopropyl Thio-Galactopiranoside) and the resistance to kanamycin. *E. coli* BL21 (DE3) was used for cloning plasmid pET29-ARA, since this strain provides the T7 polymerase required for the expression of genes controlled by PT7 promoter, and because this strain grows at high cell densities and has been used to overproduce a large number of proteins. Strain DH10B was selected for cloning of pBAD-ARA since it has been frequently used for expressing pBAD plasmids with good results.

### 2.2. Production and Extraction of the Recombinant Enzymes

**Figure 1:**
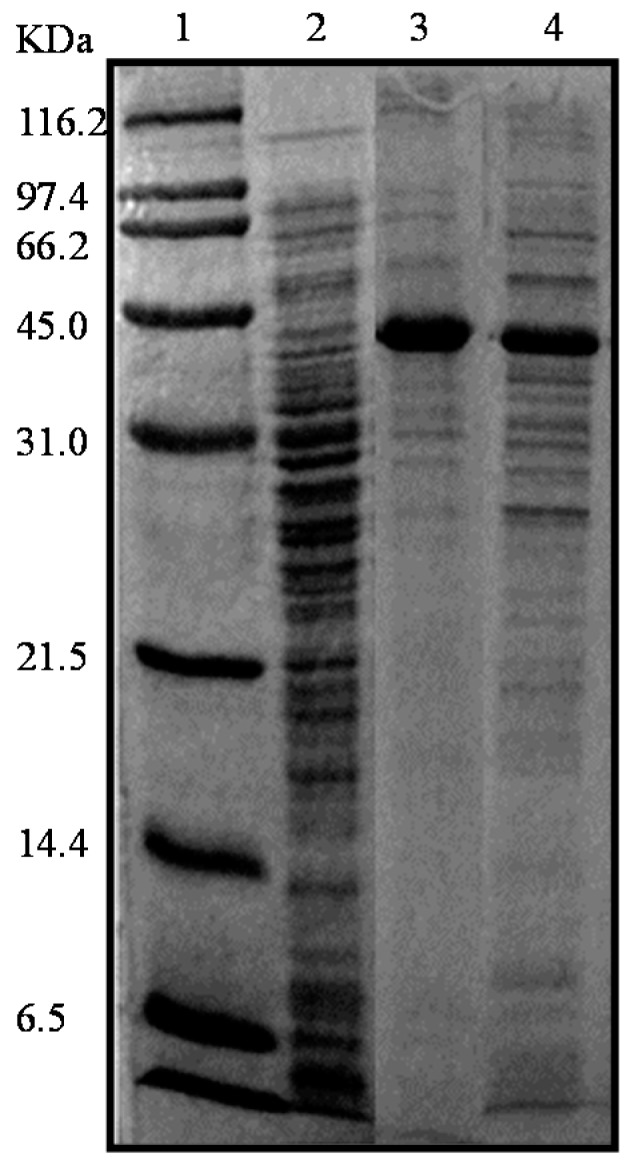
Recombinant l-AIs on Sodium Dodecyl Sulfate-PolyAcrylamide Gel Electrophoresis (SDS-PAGE) (12%). Lane 1, molecular weight standards; Lane 2, extracts of *E. coli* DH10B (pBAD-ARA) cultured without inducer; Lane 3, extracts of *E. coli* DH10B (pBAD-ARA) induced with 0.5% l-arabinose; Lane 4, extracts of *E. coli* BL21 (DE3) (pET29-ARA) induced by 1 mM IPTG (Isopropyl Thio-Galactopiranoside).

Firstly, to determine the accuracy of gene regulation in the recombinant strains to prevent the putative toxicity effect of protein overexpression, the l-AI production was performed both with and without the presence of the inducer agents, either l-arabinose or IPTG. The *E. coli* DH10B (pBAD-ARA) and *E. coli* BL21 (DE3) (pET29-ARA) cells were grown overnight in Luria Bertani medio (LB) broth at 37 °C and 200 rpm (rotations per minute) in the presence of kanamycin and ampicillin, respectively. Cells were then harvested by centrifugation and disrupted. l-AI production levels were visualized through SDS-PAGE (Sodium Dodecyl Sulfate-Polyacrylamide Gel Electrophoresis). l-AI production, meanwhile, was induced with 0.5% (*w*/*v*) l-arabinose in *E. coli* DH10B (pBDA-ARA) cells and with 1 mM IPTG in *E. coli* BL21 (DE3) (pET29-ARA) cells. As expected, enzyme expression was higher when the inducers were added (Figure 1), and the presence of a main band in the electrophoresis gel at the expected molecular weight for the enzyme monomer confirmed the success of the cloning and expression strategy. Remarkably, using both recombinant strains we have observed that most of the synthesized recombinant proteins were found in the soluble fraction. Only minor amounts of inclusion bodies were observed (data not shown). This appears to be an important and desirable characteristic of the new l-AI from *E. faecium* that behaves a highly soluble and easily folding protein in the heterologous host.

To test the production capacity of the constructions, recombinant cells were cultured in LB and Terrific Broth medio (TB), and soluble crude extracts were evaluated for l-AI activity (Table 1). We obtained the highest specific activities of l-AI in LB medium. However, as expected, the total activity was higher in the very rich TB medium. The total activity was always higher in *E. coli* DH10B (pBDA-ARA) cultures since these cultures generate more biomass than the cultures of *E. coli* BL21 (DE3) (pET29-ARA). These results suggest that both His-tagged-l-AIs are active and can be produced at very high levels in *E. coli.*

### 2.3. Purification of the Recombinant l-AIs Using Nickel and Copper Metal-Affinity Chromatography

To purify the recombinant His-tagged-l-AIs, the adsorption kinetics of both proteins were studied in two alternative immobilized metal-ion affinity chromatography (IMAC) resins, i.e., Ni-IDA-agarose and Cu-IDA-agarose. The use of two different resins allowed to determine specific the binding properties of the His-tagged enzymes and to optimize the purification protocol.

The adsorption of *N*-His-l-AI to Ni-IDA-agarose and Cu-IDA-agarose occurred within one hour, with immobilization yields close to 80% in both resins. However, the C-His-l-AI adsorption occurred more slowly because after one hour, 68% of the enzyme was adsorbed to Ni-IDA-agarose and 73% to the Cu-IDA-agarose.

**Figure 2:**
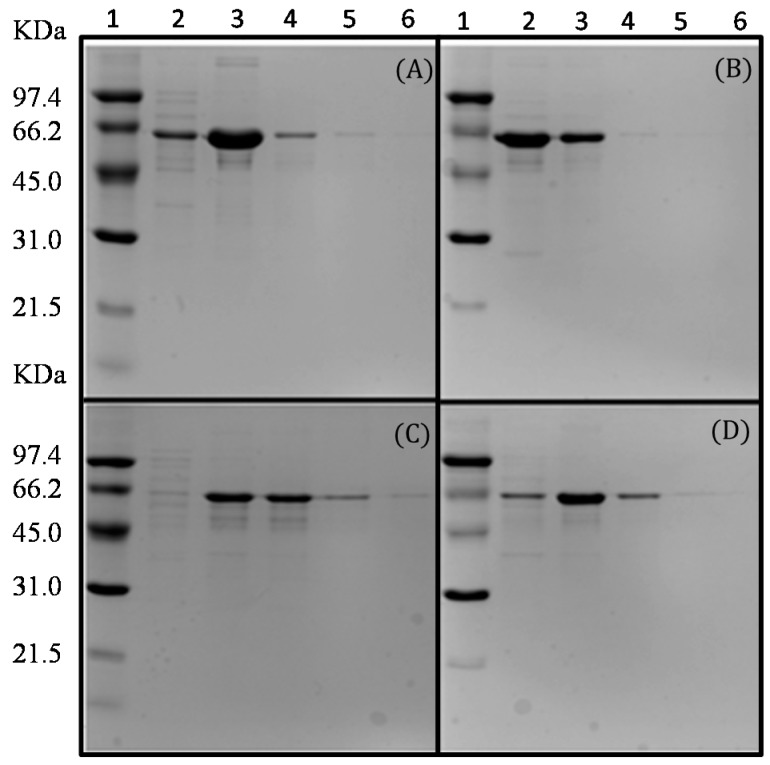
Analysis by SDS-PAGE of the purification process of the recombinant LAIs on chelated supports. Nickel- (**A**,**C**) and copper- (**B**,**D**) chelated supports. (**A**,**B**): *N*-His-l-AI. (**C**,**D**): *C*-His-l-AI. Lane 1, molecular weight standard; Lanes 2 to 6, proteins eluted with increasing concentrations of imidazole in sodium phosphate buffer 5 mM pH 7.0 (25, 50, 75, 100, 150 mM). Elutions were carried out independently for each imidazole concentration.

After their immobilization in the chelate resins both proteins were eluted at different concentrations of imidazole, and *N*-His-l-AI and *C*-His-l-AI purified on copper and nickel resins showed similar but not identical performances (Figure 2). The copper resin showed the highest retention capacity for both proteins and the *C*-His-l-AI showed higher retention capacity than the *N*-His-l-AI. Normally, this insertion does not cause significant changes in the properties of the insert. But some, such as this one, did promote some difference in the behavior of the enzyme, as a different behavior against temperature. As these changes are not intended, as a way of making sure, we have planned to make these starting changes at both ends. These changes were observed mainly when adsorption of the same on supports with chelates groups for its purification and immobilization.

### 2.4. Analysis of Recombinant l-AI Quaternary Structure by Ultracentrifugation

**Figure 3:**
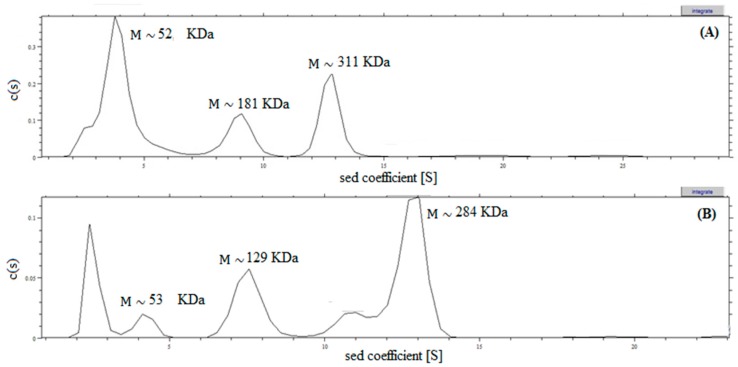
Distributions of differential sedimentation coefficient, c (S), calculated by least-squares model of the sedimentation velocity data, using SEDFIT 14.6 to determine the sedimentation coefficient (S), which is directly proportional to the mass of the particles. Analysis of the sedimentation rate corresponded to 0.43 g L^−1^ to 0.3 g L^−1^ protein, respectively, for the pure enzymes *N*-His-l-AI (**A**) and *C*-His-l-AI (**B**).

The purified proteins were used to estimate the molecular masses of the enzymes through calculation of the sedimentation coefficient (Figure 3). *N*-His-l-AI showed several peaks: (i) 3.9 S peak, compatible with the theoretical mass of the monomer with a sedimentation coefficient (S) of 3.885 × 10^−13^; (ii) 8.9 S peak, compatible with the theoretical mass of the tetramer, with a sedimentation coefficient(s) of 8.92 × 10^−13^; (iii) 12.8 S peak, compatible with the theoretical mass of the hexamer with a sedimentation coefficient(s) of 1.2777 × 10^−12^. *C*-His-l-AI also showed several peaks: (i) 4.2 S peak, compatible with the theoretical mass of the monomer, with a sedimentation coefficient (S) of 4.2020 × 10^−13^; (ii) 7.5 S peak, compatible with the theoretical mass of the trimer, with a sedimentation coefficient (s) of 7.5530 × 10^−13^ and; (iii) 12.7 S peak, compatible with the mass of the hexamer with a sedimentation coefficient (s) of 1.2740 × 10^−12^. Therefore, the predominant structures in the solution of *N*-His-l-AI were monomers (55.2%), tetramers (16.4%), and hexamers (24.2%). However, in the case of *C*-His-l-AI the predominant structures were monomers (5.3%), trimers (20.9%), and hexamers (45.2%). These results strongly suggest that both recombinant l-AIs have a tendency to form oligomers, but also suggest that the presence of the His-tag in the N- and C-terminal locations strongly influences the oligomerization. Most probably, this behavior also correlates with the different specific activities observed for both proteins. Other characterized l-AIs have tetrameric [27] or hexameric [28,29] structures.

### 2.5. Effect of pH and Temperature on the Activity and Stability of Recombinant HisTagged l-AIs 

**Figure 4:**
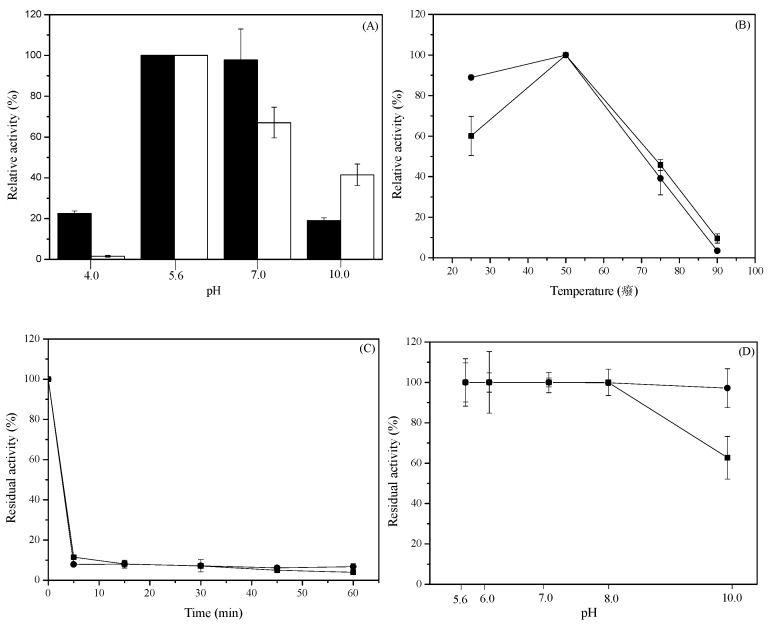
(**A**) Effect of pH on recombinant l-AI activity (■) *N*-His-l-AI, (●) *C*-His-l-AI; (**B**) Effect of temperature on the recombinant l-AIs activity. (■) *N*-His-l-AI, (●) *C*-His-l-AI; (**C**) Thermal stability of recombinant l-AIs, at 60 °C. (■) *N*-His-l-AI, (●) *C*-His-l-AI; (**D**) Effect of pH on the stability of recombinant l-AIs (■) *N*-His-l-AI, (●) *C*-His-l-AI. The enzyme samples were incubated at different pH buffering systems (5.6; 6.0; 7.0; 8.0; 10.0) at 50 mM. The reactions were performed in 50 mM sodium acetate buffer pH 5.6.

The highest l-AI activity was achieved at pH 5.6 at 50 °C with 400 mM of d-galactose and 1 mM MnCl_2_. Maximal activities of *N*-His-l-AI and *C*-His-l-AI at pH 5.6 were 2.77 ± 0.068 U mL^−1^ and 1.86 ± 0.069 U mL^−1^, respectively. *N*-His-l-AI showed a high enzymatic activity within pH 5.6–7.0. However, *C*-His-l-AI enzyme presented a constant decrease in activity when pH was increased from 5.6 to 10. This behavior could be explained by the effect caused by the position of His-tag. The acidophilic preference of *E. faecium* l-AI was also observed in l-AIs from other organisms, e.g., *Lactobacillus gayonii* [30], *Shewanella* sp. ANA-3 [27], *Bifidobacterium longum* NRRL B-41409 [31], and *Alicyclobacillus acidocaldarius* [17]. A low catalytic activity was observed at pH 10 with *N*-His-l-AI, i.e., only 19% of the activity observed at pH 5.6; *C*-His-l-AI, however, retained 41% of the activity at pH 10 (Figure 4A). These results agree with the finding that the optimal pH value for the native l-AI was around pH 7.0 [11], but also suggest that the presence of the His-tag slightly modifies the behavior of the enzyme at different pHs.

Acidic environments are more favorable and advantageous for d-tagatose production due to the lower generation of by-products, becoming a more attractive source for their use in industrial and biotechnological applications [17,32]. In addition, neutral pH is not recommended for the isomerization reaction because an undesirable darkening and reduced-sweetness of the produced-carbohydrates is caused by the Maillard reaction [33].

The optimum temperature for enzyme activity for both His-tagged enzymes was 50 °C at pH 5.6. *C*-His-l-AI enzyme showed a broad optimum temperature range of 25–50 °C (Figure 4B). Maximal activities of *N*-His-l-AI and *C*-His-l-AI were 2.76 ± 0.16 U mL^−1^ and 1.34 ± 0.09 U mL^−1^, respectively. When thermostability experiments were conducted with both recombinant enzymes at 60 °C we observed a fast decrease in enzyme activity, with the enzymes almost fully inactivated after 5 min of incubation (Figure 4C).

The effect of pH on the stability of recombinant l-AIs was investigated by incubating the enzymes at room temperature under constant agitation with various buffer systems of different pH. The stability of *N*-His-l-AI decreased to approximately 37% at pH 10.0, but *C*-His-l-AI showed only a 3% reduction of activity at this pH (Figure 4D).

### 2.6. d-Tagatose Conversion by Recombinant l-AIs in Solution

**Figure 5:**
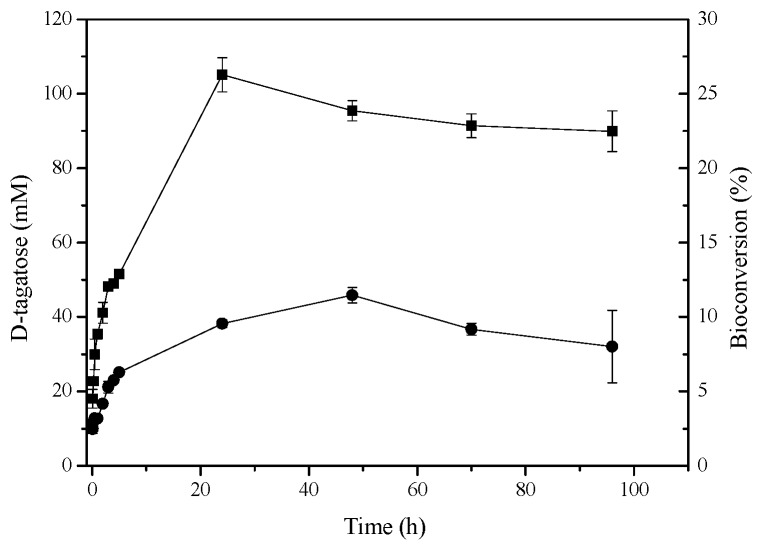
Enzymatic synthesis of d-tagatose catalyzed by recombinant l-AIs. (■) *N*-His-l-AI (●) *C*-His-l-AI in sodium acetate buffer (pH 5.6), with 1 mM MnCl_2_ as cofactor, at 50 °C. Product concentration and substrate conversion are represented as a function of the reaction time. Initial concentration of d-galactose was 400 mM. Reaction was carried out in a final volume of 2 mL using 2.02 units of *N*-His-l-AI or 1.05 units of *C*-His-l-AI.

The maximum concentrations of d-tagatose obtained from d-galactose using *N*-His-l-AI and *C*-His-l-AI were 105 and 46 mM, respectively, representing a 26% conversion yield in 24 h for *N*-His-l-AI and an 11% conversion yield in 48 h for *C*-His-l-AI (Figure 5). The bioconversion yield of d-galactose into d-tagatose for other recombinant l-AIs ranges between 16% and 40% [12,22,27].

### 2.7. Determination of the Kinetic Models for Both Recombinants l-AIs Using d-Galactose As Substrate

**Figure 6:**
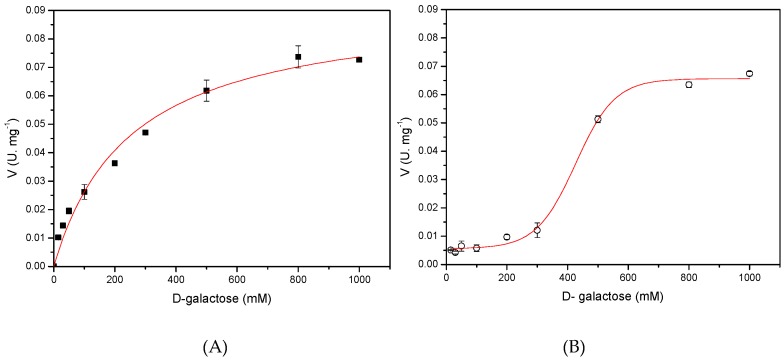
Influence of d-galactose concentration in the initial rate of isomerization reaction catalyzed by the recombinant l-AIs. (**A**, ■) *N*-His-l-AI, (**B**, ○) *C*-His-l-AI. Reactions were carried out in sodium acetate buffer (pH 5.6), with 1 mM MnCl_2_ as cofactor, at 50 °C in a final volume of 0.5 mL using 3.0 mg of *N*-His-l-AI or 3.9 mg of *C*-His-l-AI. The lines represent a hyperbolic model for *N*-His-l-AI and a sigmoidal model for *C*-His-l-AI using Origin 8.1 software.

The kinetic mechanism of the recombinant l-AIs was studied using d-galactose as substrate by measuring the initial velocities of the enzymatic reaction. Results revealed that *N*-His-l-AI enzyme follows a Michaelis-Menten kinetic (Figure 6A). In contrast, *C*-His-l-AI enzyme fitted to a cooperative interaction model between the enzyme monomers, which was represented as a sigmoidal saturation curve (Figure 6B). This latter tendency is common in multimeric enzymes such as l-AI, where a conformational change in enzyme structure is usually evidenced [34]. After fitting the curves in Figure 6, kinetic parameters Km and Vmax for the recombinant enzymes were determined. *N*-His-l-AI showed a Km of 252 mM and a Vmax of 0.092 U mg^−1^, whereas *C*-His-l-AI showed a K_0.5_ of 426 mM and a Vmax of 0.066 U mg^−1^. The affinity of l-AI to the substrate d-galactose varies with the source of enzyme [35,36], and our results suggest that the position of the His-tag causes structural changes in the recombinant l-AIs that differentially affect their catalytic parameters.

## 3. Materials and Methods 

### 3.1. Materials 

d-Tagatose, d-galactose, l-arabinose, l-cysteine, epichlorohydrin, iminodiacetic acid (IDA), sodium periodate (NaIO_4_), isopropyl-1-thio-β-d-galactopyranoside (IPTG), ampicillin, kanamycin, carbazole crystalline, and agarose CL-4B were purchased from Sigma (Sigma-Aldrich, St. Louis, MO, USA). In addition, different analytical grade reagents from different trademarks were used.

### 3.2. Genetic Engineering Procedures

The sequence of the *araA* gene of *E. faecium* DBFIQ E36 (Cátedras de Microbiología y Biotecnología, Departamento de Ingeniería en Alimentos, Facultad de Ingeniería Química (FIQ), Universidad Nacional del Litoral (UNL), Santa Fe, Argentina collection) previously identified by Manzo et al. [15] was used for cloning purposes. The codon usage of the *araA* gene was optimized in silico for its expression in *E. coli* according to the software provided at IDT (Integrate DNA Technology, Inc., San Francisco, CA, USA) [37]. Using the optimized *araA* sequence we designed two synthetic genes to produce two different His-tagged l-AIs containing six histidines at its N- and C-terminal ends, respectively. The expression of both chimeric genes was tested using two different inducible promoters and two different *E. coli* strains.

### 3.3. Synthesis of the Gene Encoding the N-Terminal His-Tagged l-AI. Expression under the Control of the ParaC-Inducible Promoter

The gene encoding the N-terminal His-tagged l-AI (*N*-His-l-AI) was constructed by replacing the ATG (Adenosine, Thymine, and Guanosine) initiation codon of the *araA* gene from *E. faecium* DBFIQ E36 with the sequence CCATGGGTCATCATCATCATCACCAT, and the TAA codon with the sequence TAATAGAATTC. The first sequence provides an NcoI restriction site, the ATG codon, a GGT codon (coding for glycine in order to improve its translation) and 6 His codons. The second sequence provides an additional in-tandem stop codon and an EcoRI restriction site. The new codon-optimized chimeric *araA* gene containing these additional sequences was synthesized by ATG: Biosynthetics GmbH (Dusseldorf, Germany). The synthetic gene was cut with NcoI and EcoRI and cloned into the equivalent restriction sites of the pBAD/HisA vector (Invitrogen, Carlsbad, CA, USA) to generate the plasmid pBAD-ARA, arabinose inducible expression, which was transformed into *E. coli* DH10B cells by electroporation. The recombinant plasmid was sequenced to confirm the construction. The resulting recombinant *N*-His-l-AI contains 481 aa (amino acids) including the initial methionine (MW 55,036). The production of *N*-His-l-AI using the pBAD-ARA vector is controlled by the ParaC promoter, which is inducible by l-arabinose.

### 3.4. Synthesis of the Gene Encoding the C-Terminal His-Tagged l-AI. Expression Under the Control of PT7—Inducible Promoter

To construct the gene encoding the C-terminal His-tagged l-AI (*C*-His-l-AI), we replaced the ATG codon of the *araA* gene from *E. faecium* DBFIQ E36 with the sequence TCTAGAAAT AATTTTGTTTAACTTTAAGAAGGAGATATACATATG and the TAA codon with the sequence CTCGAGTAA. The first sequence provided an XbaI restriction site, the long Shine-Dalgarno sequence used for PT7-expressed genes and the ATG codon. The second sequence provided an XhoI restriction site and the TAA stop codon. The new codon-optimized chimeric *araA* gene containing these additional sequences was synthesized by ATG. The synthetic gene was cut with XbaI and XhoI and cloned into the equivalent sites of the pET29a vector (Novagen, Darmstadt, Germany) to generate the plasmid pET29-ARA, which was transformed into *E. coli* BL21 (DE3) cells by electroporation. After cloning the synthetic gene into the plasmid pET29a, the resulting gene contains at its 3′-end the 6 His-tag coding sequence provided by the plasmid. The recombinant plasmid was sequenced to confirm the construction. The resulting recombinant *C*-His-l-AI contains 482 aa including the initial methionine (MW 55,221). The production of *C*-His-l-AI using the pET29-ARA vector is controlled by the PT7 promoter that is inducible by IPTG.

### 3.5. Enzyme Production

*E. coli* recombinant cells were usually cultured at 37 °C in LB media (Difco brand) containing ampicillin (100 μg mL^−1^) for *E. coli* DH10B (pBAD-ARA) or kanamycin (50 μg mL^−1^) for *E. coli* BL21 (DE3) (pET29-ARA) [38]. For enzyme production, cultures were induced at a DO 600 nm 0.7–0.9 with 1 mM IPTG in the case of *E. coli* BL21 (DE3) (pET29-ARA) or 0.5% (*w*/*v*) l-arabinose in the case of *E. coli* DH10B (pBAD-ARA). The induced cells were incubated for 12 h at 37 °C and 200 rpm orbital agitation speed. Cells were then harvested by centrifugation at 6000× *g* for 20 min at 4 °C and resuspended in 50 mM sodium phosphate buffer (pH 7.0) and disrupted by a French Press at 1100 lb/in^2^. After disruption, cell debris was removed by centrifugation at 15,000× *g* for 20 min at 4 °C [38]. The protein content of the supernatant (crude extract) was analyzed by SDS-PAGE (see below). To increase cell biomass some experiments were also performed using TB [39] instead of LB medium. In this case, the inducers were added when the culture reached a DO 600 nm of 2.0.

### 3.6. Protein Analyses

Protein concentration was determined by Coomassie Blue G-250 with BSA (bovine serum albumin), (Sigma-Aldrich, Madrid, Spain) as standard [40]. Proteins were also analyzed by SDS-PAGE [41] using a Bio-Rad (model Mini-PROTEAN 3 Cell, 1000 Alfred Nobel Drive, Hercules, CA, USA) equipment with a Bio-Rad Power Pac Basic power supply. SDS-PAGE low-molecular-weight standards (14.4–97.0 kDa; GE Healthcare, Little Chalfont, UK) were used for molecular weight estimation.

### 3.7. l-AI Assay

l-AI assay was performed with 400 mM d-galactose as substrate prepared in 50 mM sodium acetate buffer (pH 5.6) and supplemented with 1 mM MnCl_2_. The samples were incubated at 50 °C and the amount of d-tagatose was determined by measuring the absorbance at 560 nm via the cysteine-carbazole sulfuric acid method [42]. One unit (U) of l-AI activity was defined as the amount of enzyme required for the production of one µmol of d-tagatose per minute under the reaction conditions [12]. The influence of pH on the activity of l-AI was determined employing 50 mM sodium acetate buffer (pH 5.6), 50 mM sodium phosphate buffer (pH 7.0), and 50 mM sodium bicarbonate buffer (pH 10).

Optimum temperature for l-AI activity was determined in 50 mM sodium phosphate buffer (pH 7.0) at temperatures ranging from 25 °C to 90 °C. To determine pH stability of l-AI, the enzyme was incubated in different buffers at room temperature for two hours at 50 mM final concentration. The conditions assayed were in sodium acetate buffer (pH 5.6), sodium phosphate buffer (pH 6.0, 7.0 and 8.0), and sodium bicarbonate buffer (pH 10.0) with the addition of 1 mM MnCl_2_ as cofactor in all cases. The thermal stability of l-AI was studied by determining the remaining enzyme activity after incubation of the enzyme at 60 °C, over 60 min. Samples were periodically withdrawn at different time intervals and immediately placed into a cold ice bath (0 °C) before being assayed as described above. Relative activity curves as a function of time were elaborated using Origin 8.1. 

### 3.8. Preparation of Affinity Supports

Epoxy-activated agarose supports were obtained using epichlorohydrin as previously described [43]. For this purpose, 100 g dried-filtered agarose CL-4B were resuspended in 440 mL of distilled water and, in the following order, 160 mL of acetone, 32.8 g of NaOH, 2 g of NaBH_4_ and 110 mL of epichlorohydrin were added. The suspension was gently stirred for 16 h and then rinsed with an excess of water. The preparation of IDA (Immino Diacetic Acid) supports was carried out as described [38]. Activated-agarose (20 g) was suspended in 200 mL of 0.5 M IDA prepared in 0.1 M sodium bicarbonate buffer (pH 11). The solution pH was maintained around 11 and the preparation was gently stirred at 25 °C for 12 h. Subsequently, the IDA support was filtered and rinsed with distilled water and immediately vacuum-dried. The preparation of the metal chelate bioadsorbent was performed by incubating the IDA derivatives in Milli-Q water containing either 5 mg/mL of CuSO_4_ (support/solution ratio of 10 g of IDA support/100 mL CuSO_4_ solution) or 50 mM sodium phosphate buffer (pH 6.0) containing 1.0 M NaCl plus 5 mg mL^−1^ of NiCl_2_ (support/solution/salt ratio of 10 g of IDA support/100 mL NiCl_2_ solution/0.1 g NaCl) and gently stirred at 25 °C for 2 h [44]. Finally, the supports were washed thoroughly with distilled water and vacuum-dried.

### 3.9. Purification of the Recombinant l-AI by Metal-Affinity Chromatography

The recombinant His-tagged l-AIs were purified through IMAC using Ni^2+^ or Cu^2+^ as alternative metal ions for chelation with IDA supports. To adsorb the enzyme to the supports, 1 g of each metal chelate support was added to 10 mL of clarified recombinant l-AI crude extract prepared in 50 mM sodium phosphate buffer (pH 7.0) with the addition of 150 mM NaCl. Enzyme was attached to the bioadsorbents at room temperature with constant stirring, and a control experiment for the assessment of probable recombinant enzyme deactivation under the same enzyme binding conditions as in purification assays was carried out. The adsorbed proteins were eluted with aliquots of 5 mL of increasing concentrations of imidazole (25, 50, 75, 100, 150, 200, 300, 350, and 400 mM) after 30 min incubation time for each concentration. Proteins were analyzed by 12.5% SDS-PAGE according to the methodology proposed above. l-AI activity was measured spectrophotometrically as described above. Purification steps were performed at room temperature [38].

### 3.10. Estimation of Kinetic Parameters

The initial rates of conversion of d-galactose to d-tagatose were determined at 50 °C in 50 mM sodium acetate buffer (pH 5.6) with the addition of 1 mM MnCl_2_. For this purpose, substrate concentrations of 15, 30, 50, 100, 200, 300, 500, 800 and 1000 mM of d-galactose were used. Assuming a Michaelis-Menten approach, kinetic parameters (Km and Vmax) were calculated by non-linear fit of the initial rates in function of substrate concentration using Origin 8.1.

### 3.11. Bioconversion Assays

To determine the percentage of bioconversion of d-galactose to d-tagatose, l-AI enzyme was incubated at 50 °C for 96 h with 400 mM of d-galactose in 50 mM sodium acetate buffer (pH 5.6), with the addition of a final concentration of 1 mM MnCl_2_. Samples were taken at different time intervals and reaction was stopped by chilling on ice. The amount of d-tagatose was then determined as described [42].

### 3.12. Analytical Ultracentrifugation

Sedimentation velocity experiments were performed using an An-50Ti rotor 72,446× *g* at 20 °C in a XLI analytical ultracentrifuge (Beckman-Coulter Inc., Life Sciences Division Headquarters 5350, Indianapolis, CA, USA) equipped with UV/VIS absorbance detector employing a wavelength of 285 nm. Three concentrations of the purified recombinant proteins were tested, i.e., 0.1; 0.22; 0.43 g L^−1^ for *N*-His-l-AI and 0.1; 0.15; 0.3 g L^−1^ for *C*-His-l-AI. The proteins were diluted and equilibrated in 50 mM sodium phosphate buffer (pH 7.0). The distribution of the sedimentation coefficient, c(s), was calculated by least-squares regression of the achieved sedimentation velocity data using the computer program SEDFIT 14.6e [45]. 

## 4. Conclusions

In this work, the l-AI from *E. faecium* DBFIQ E36 was produced in *E. coli* in two different His-tagged forms (N-terminal and C-terminal tagged proteins), resulting in satisfactory amounts of both recombinant l-AIs in the soluble forms using two different expression systems and two different *E. coli* hosts. As expected, both His-tagged-l-AIs can be purified by metal-affinity chromatography in a single step. However, all the characteristics determined for these two proteins suggest that the presence of the His-tag at the N- or C-terminal ends of the l-AI is not trivial because the tag promotes significant and different changes in their enzymatic and structural properties. Both recombinant l-AIs produced in this work showed different characteristics that are promising for their application on an industrial scale.

## Figures and Tables

**Table 1:** Activity of cell extracts enriched l-AI produced in *E. coli* DH10B (pBAD-ARA) and BL21 (DE3) (pET29-ARA), in LB and TB media.

	Extracts	Activity (U mL^−1^)	Protein Concentration (mg mL^−1^)	Specific Activity (U mg^−1^)
**Luria Bertani (LB) medium**	*N*-His-l-AI	6.21	40	0.154
	*C*-His-l-AI	3.01	31	0.097
**Terrific Broth (TB) medium**	*N*-His-l-AI	10.09	298	0.034
	*C*-His-l-AI	5.29	145	0.036

## References

[1] Choi J.H., Chung S.J. (2015). Sweetness potency and sweetness synergism of sweeteners in milk and coffee systems. Food Res. Int..

[2] Hong Y.H., Lee D.W., Lee S.J., Choe E.A., Kim S.B., Lee Y.H., Cheigh C.I., Pyun Y.R. (2007). Production of d-tagatose at high temperatures using immobilized *Escherichia coli* cells expressing l-arabinose isomerase from *Thermotoga neapolitana*. Biotechnol. Lett..

[3] Xu Z., Li S., Feng X., Liang J., Xu H. (2014). l-Arabinose isomerase and its use for biotechnological production of rare sugars. Appl. Microbiol. Biotechnol..

[4] Grant L.D., Bell L.N. (2012). Physical and chemical stability of tagatose powder. J. Food Sci..

[5] Rouhi M., Mohammadi R., Mortazavian A.M., Sarlak Z. (2015). Combined effects of replacement of sucrose with d-tagatose and addition of different probiotic strains on quality characteristics of chocolate milk. Dairy Sci. Technol..

[6] Boudebbouze S., Maguin E., Rhimi M. (2011). Bacterial l-arabinose isomerases: Industrial application for d-tagatose production. Recent Pat. DNA Gene Seq..

[7] Ghizalba O., Meyer H.P., Wohlgemuth R., Flickinger M.C. (2010). Industrial Biotransformation. Encyclopedia of Industrial Biotechnology: Bioprocess, Bioseparation, and Cell Technology.

[8] Sheldon R.A., van Pelt S. (2013). Enzyme immobilisation in biocatalysis: Why, what and how. Chem. Soc. Rev..

[9] Oh D.K. (2007). Tagatose: Properties, applications, and biotechnological processes. Appl. Microbiol. Biot..

[10] Liang M., Chen M., Liu X., Zhai Y., Liu X.W., Zhang H., Xiao M., Wang P. (2012). Bioconversion of d-galactose to d-tagatose: Continuous packed bed reaction with an immobilized thermostable l-arabinose isomerase and efficient purification by selective microbial degradation. Appl. Microbiol. Biotechnol..

[11] Torres P.R., Manzo R.M., Rubiolo A.C., Batista-Viera F.D., Mammarella E.J. (2014). Purification of an l-arabinose isomerase from Enterococcus faecium DBFIQ E36 employing a biospecific affinity strategy. J. Mol. Catal. B Enzym..

[12] Hung X.G., Tseng W.C., Liu S.M., Tzou W.S., Fang T.Y. (2014). Characterization of a thermophilic l-arabinose isomerase from Thermoanaerobacterium saccharolyticum NTOU1. Biochem. Eng. J..

[13] Men Y., Zhu Y., Zhang L., Kang Z., Izumori K., Sun Y., Ma Y. (2014). Enzymatic conversion of d-galactose to d-tagatose: Cloning, overexpression and characterization of l-arabinose isomerase from Pediococcus pentosaceus PC-5. Microbiol. Res..

[14] Lima A.F., Cavalcante K.F., de Freitas M.F.M., Rodrigues T.H.S., Rocha M.V.P., Gonçalves L.R.B. (2013). Comparative biochemical characterization of soluble and chitosan immobilized β-galactosidase from Kluyveromyces lactis NRRL Y1564. Process Biochem..

[15] Manzo R.M., Simonetta A.C., Rubiolo A.C., Mammarella E.J. (2013). Screening and selection of wild strains for l-arabinose isomerase production. Braz. J. Chem. Eng..

[16] Lee D.W., Choe E.A., Kim S.B., Eom S.H., Hong Y.H., Lee S.J., Lee H.S., Lee D.Y., Pyun Y.R. (2005). Distinct metal dependence for catalytic and structural functions in the l-arabinose isomerase from the mesophilic Bacillus halodurans and the thermophilic Geobacillus stearothermophilus. Arch. Biochem. Biophys..

[17] Lee S.J., Lee D.W., Choe E.A., Hong Y.H., Kim S.B., Kim B.C., Pyun Y.R. (2005). Characterization of a thermoacidophilic l-arabinose isomerase from Alicyclobacillus acidocaldarius: Role of Lys-269 in pH optimum. Appl. Environ. Microbiol..

[18] Zhang H., Jiang B., Pan B. (2007). Purification and characterization of l-arabinose Isomerase from Lactobacillus plantarum producing d-tagatose. World J. Microb. Biotechnol..

[19] Xu Z., Li S., Fu F., Li G., Feng X., Xu H., Ouyang P. (2012). Production of d-tagatose, a functional sweetener, utilizing alginate immobilized *Lactobacillus fermentum* CGMCC2921 cells. Appl. Biochem. Biotechnol..

[20] Zhan Y., Xu Z., Li S., Liu X., Xu L., Feng X., Xu H. (2014). Coexpression of β-d-galactosidase and l-arabinose isomerase in the production of d-tagatose: A functional sweetener. J. Agric. Food Chem..

[21] Cheon J., Kimb S.B., Parkb S.W., Hanb J.K., Kima P. (2009). Characterization of l-Arabinose isomerase in *Bacillus subtilis*, a gras host, for the production of edible tagatose. Food Biotechnol..

[22] Staudigl P., Haltrich D., Peterbauer C.K. (2014). l-Arabinose isomerase and d-xylose isomerase from *Lactobacillus reuteri*: Characterization, coexpression in the food grade host *Lactobacillus plantarum*, and application in the conversion of d-galactose and d-glucose. J. Agric. Food Chem..

[23] Lee D.W., Jang H.J., Choe E.A., Kim B.C., Lee S.J., Kim S.B., Hong Y.H., Pyun Y.R. (2004). Characterization of a thermostable l-arabinose (d-galactose) isomerase from the hyperthermophilic eubacterium *Thermotoga maritima*. Appl. Environ. Microbiol..

[24] Zhou X., Wu J.C. (2012). Heterologous expression and characterization of *Bacillus coagulans*
l-arabinose isomerase. World J. Microbiol. Biotechnol..

[25] Kim J.W., Kim Y.W., Roh H.J., Kim H.Y., Cha J.H., Park K.H., Park C.S. (2003). Production of tagatose by a recombinant thermostable l-arabinose isomerase from *Thermus* sp. IM6501. Biotechnol. Lett..

[26] Rhimi M., Messaoud E.B., Borgi M.A., Khadra K.B., Bejar S. (2007). Co-expression of l-arabinose isomerase and d-glucose isomerase in *E. coli* and development of an efficient process producing simultaneously d-tagatose and d-fructose. Enzym. Microb. Technol..

[27] Rhimi M., Bajic G., Ilhammami R., Boudebbouze S., Maguin E., Haser R., Aghajari N. (2011). The acid-tolerant l-arabinose isomerase from the mesophilic *Shewanella* sp. ANA-3 is highly active at low temperatures. Microb. Cell Fact..

[28] Patrick J.W., Lee N. (1969). Subunit Structure of Arabinose Isomerase from *Escherichia coli*. J. Biol. Chem..

[29] Choi J.M., Lee Y.J., Cao T.P., Shin S.M., Park M.K., Lee H.S., Luccio E., Kim S.B., Lee S.J., Lee S.J. (2016). Structure of the thermophilic l-Arabinose isomerase from *Geobacillus kaustophilus* reveals metal-mediated intersubunit interactions for activity and thermostability. Arch. Biochem. Biophys..

[30] Nakamatu T., Yamanaka K. (1969). Crystallization and properties of l-arabinose isomerase from *Lactobacillus gayonii*. Biochim. Biophys. Acta Enzymol..

[31] Salonen N., Nyyssölä A., Salonen K., Turunen O. (2012). Bifidobacterium longum l-arabinose isomerase—Overexpression in *Lactococcus lactis*, purification, and characterization. Appl. Biochem. Biotechnol..

[32] Rhimi M., Juy M., Aghajari N., Haser R., Bejar S. (2007). Probing the essential catalytic residues and substrate affinity in the thermoactive Bacillus stearothermophilus US100 l-arabinose isomerase by site-directed mutagenesis. J. Bacteriol..

[33] Liu S.Y., Wiegel J., Gherardini F.C. (1996). Purification and cloning of a thermostable xylose (glucose) isomerase with an acidic pH optimum from *Thermoanaerobacterium* strain JW/SL-YS 489. J. Bacteriol..

[34] Ricard J.A. (1987). Cornish-Bowden A. Co-operative and allosteric enzymes: 20 Years on. Eur. J. Biochem..

[35] Ibrahim O.O., Spradlin. J.E. (2000). Process for Manufacturing d-tagatose. U.S. Patent.

[36] Rhimi M., Bejar S. (2006). Cloning, purification and biochemical characterization of metallic-ions independent and thermoactive l-arabinose isomerase from the *Bacillus stearothermophilus* US100 strain. Biochim. Biophys. Acta.

[37] Codon Optimization—Integrated DNA Technologies. http://eu.idtdna.com/CodonOpt.

[38] Pessela B.C.C., Vian A., Mateo C., Lafuente R., García J.L., Guisan J.M., Carrascosa A.V. (2003). Overproduction of *Thermus* sp. strain T2 beta-galactosidase in *Escherichia coli* and preparation by using tailor-made metal chelate supports. Appl. Environ. Microb..

[39] Sambrook J.F., Russell D. (2001). Molecular Cloning: A Laboratory Manual.

[40] Bradford M.M. (1976). A rapid and sensitive method for the quantitation of microgram quantities of protein utilizing the principle of protein-dye binding. Anal. Biochem..

[41] Laemmli U.K. (1970). Cleavage of structural protein during the assembly of the heat of bacteriophage T4. Nature.

[42] Dische Z., Borenfreund E. (1951). A new spectrophotometric method for the detection and determination of keto sugars and trioses. J. Biol. Chem..

[43] Bolívar J.M., Rocha-Martin J., Godoy C., Rodrigues R.C., Guisán J.M. (2010). Complete reactivation of immobilized derivatives of a trimeric glutamate dehydrogenase from *Thermus thermophillus*. Process Biochem..

[44] Armisén P., Mateo C., Cortés E., Barredo J.L., Salto F., Diez B., Rodés L., García J.L., Fernández-Lafuente R., Guisan J.M. (1999). Selective adsorption of poly-His tagged glutaryl acyiase on tailor-made metal chelate supports. J. Chomatogr. A.

[45] Pessela B.C.C., Mateo C., Carrascosa A.V., Vian A., García J.L., Rivas G., Alfonso C., Guisan J.M., Fernandez-Lafuente R. (2003). One-step purification, covalent immobilization, and additional stabilization of a thermophilic poly-His tagged‚ β-galactosidase from *Thermus* sp. Strain T2 by using novel heterofunctional chelate-epoxy sepabeads. Biomacromolecules..

